# #BoomerRemover: COVID-19, Ageism, and the Intergenerational Twitter Response

**DOI:** 10.1093/geroni/igaa057.3414

**Published:** 2020-12-16

**Authors:** Antonius Skipper, Daniel Rose

**Affiliations:** 1 Georgia State University, Atlanta, Georgia, United States; 2 Winston-Salem State University, Winston-Salem, North Carolina, United States

## Abstract

In March 2020, COVID-19 was declared a pandemic and frequently presented as a virus primarily affecting older adults. News headlines led with statements such as, “Coronavirus deaths are so far mostly older men” (Ramzy, 2020). Although later determined inaccurate, this perspective contributed to openly ageist views and exchanges from people around the world. On the social media platform of Twitter, #BoomerRemover was used as a hashtag to express views related to older adults, and particularly baby boomers, as the primary targets of COVID-19. This study uses qualitative methods to analyze the messages of Twitter users that discuss COVID-19 with the use of the hashtag #BoomerRemover. A total of 1,875 tweets posted in English and including the hashtag “#BoomerRemover” from March 16, 2020 to March 30, 2020 were analyzed. Analytic methods employed an open coding procedure consistent with grounded theory and Numeric Content Analysis (Marks, 2015). Salient themes include: (1) COVID-19 is Politically Driven (2) There’s a Real Intergenerational Divide, (3) Young People are Dying Too, and (4) #BoomerRemover is Simply Disrespectful. Findings suggest that only about a fourth of #BoomerRemover tweets could be considered ageist, and the large majority of tweets using the hashtag were related to politics and elections. Further, several of those using the #BoomerRemover hashtag to defend older adults were inadvertently causing it to remain relevant (trend) as a Twitter topic. This study recognizes the importance of considering Twitter – primarily composed of young adults – as a place where intergenerational attitudes vis-à-vis COVID-19 may be expressed.

